# Application of the ATP assay to rapidly assess cleanliness of spacecraft surfaces: a path to set a standard for future missions

**DOI:** 10.1186/s13568-016-0286-9

**Published:** 2016-11-14

**Authors:** James N. Benardini, Kasthuri Venkateswaran

**Affiliations:** Biotechnology and Planetary Protection Group, Jet Propulsion Laboratory, California Institute of Technology, 4800 Oak Grove Dr., M/S 89-2, Pasadena, CA 91109 USA

**Keywords:** Planetary protection, Spore, Burden, ATP, NASA standard assay, MSL

## Abstract

The National Aeronautics and Space Administration (NASA) measures and validates the biological cleanliness of spacecraft surfaces by counting endospores using the NASA standard assay (NSA). NASA has also approved an adenosine-5′-triphosphate (ATP)-based detection methodology as a means to prescreen surfaces for the presence of microbial contamination, prior to the spore assay. During Mars Science Laboratory (MSL) spacecraft assembly, test, and launch operations, 4853 surface samples were collected to verify compliance with the bioburden requirement at launch. A subset of these samples was measured for microbial cleanliness using both the NSA (n = 272) and ATP assay (n = 249). NSA results revealed that ~8% (22/272) of the samples showed the presence of at least one spore, whereas ATP assay measurements indicated that ~15% (35/249) of samples exceeded the “threshold cleanliness limit” of 2.3 × 10^−11^ mmol ATP per 25 cm^2^ used by MSL. Of the 22 NSA samples with a spore, 18% (4/22) were considered above the level of acceptance by both techniques. Based on post launch data analysis presented here, it was determined that this threshold cleanliness limit of 2.3 × 10^−11^ mmol ATP per 25 cm^2^ could be adopted as a benchmark for assessing spacecraft surface cleanliness. This study clearly demonstrates the value of using alternative methods to rapidly assess spacecraft cleanliness, and provides useful information regarding the process.

**Electronic supplementary material:**

The online version of this article (doi:10.1186/s13568-016-0286-9) contains supplementary material, which is available to authorized users.

## Introduction

The National Aeronautics and Space Administration (NASA) has an obligation to comply with international policy for missions to Mars by not jeopardizing possible extraterrestrial life forms, precursors, and remnants through the conduct of scientific investigations (COSPAR 2011). Traditionally, NASA measures spacecraft surface biological cleanliness by counting endospores, since they are most often the predominant survivors of dry heat microbial reduction flight hardware processing and have been reported to survive in extreme conditions (Favero 1971; La Duc et al. 2004; NASA 2011; Puleo et al. 1977). Since counting endospores using the NASA standard assay (NSA) is time consuming, requiring a 72 h incubation period, a rapid and sensitive adenosine-5′-triphosphate (ATP)-based molecular assay, requiring only a minute for sample processing, has been approved by NASA as a means to screen spacecraft hardware for the presence of microbial contamination (Kern et al. 2005; Morris et al. 2010). This methodology (hereafter referred to as the ATP assay), which measures ATP associated with living and dead microbes, has not currently been approved by NASA as an alternative to the spore assay for determining overall microbial contamination of spacecraft.

When the ATP assay methodology was first validated using the Mars Exploration Rovers as a testbed (MER-ATP), it was understood that there could be no direct correlation observed between bacterial spores, as measured by the NSA, and biomarkers, as measured by the ATP assay. The ATP assay, however, was recommended as a suitable methodology for monitoring hardware being brought into the controlled, clean room spacecraft assembly environment. It was also suggested that the ATP assay was appropriate for assessing the microbial cleanliness of component surfaces before they were mated (Kern et al. 2005). The MER-ATP study recommended that if spacecraft surfaces were found to be at a threshold cleanliness limit of ≤2.57 × 10^−11^ ATP mmoles per 25 cm^2^ by the ATP assay, but spore count was zero by the NSA, the sampled surface should be considered clean for spore accounting purposes (Kern et al. 2005). (In the MER-ATP study’s field tests, when aliquots of the same collected samples were examined with both the ATP assay and NSA, no such discrepancies were actually observed.) The MER-ATP study also recommended a higher threshold, or control level, for required recleaning (≥3.51 × 10^−11^ ATP mmoles per 25 cm^2^). Subsequently, NASA adopted these threshold values and approach as an accepted supplemental spacecraft cleanliness assessment assay to the NSA (NASA 2010).

NASA’s largest rover to date, the Mars Science Laboratory (MSL), was launched in November 2011 to assess the habitability for past life, investigate the surface geology, and characterize the radiation environment of Mars. MSL was assembled to meet stringent microbial cleanliness requirements that involved contamination controls including clean room assembly and microbial reduction (COSPAR 2011; NASA 2011). Since NASA had approved the ATP assay to prescreen spacecraft hardware for the presence of microorganisms prior to conducting the NSA, the MSL project adopted it for this purpose and used it as a contamination risk reduction tool. MSL was the first NASA mission that utilized the ATP assay to measure hardware cleanliness for mandatory inspection points, while using the NSA for official spore count enumeration.

Based on the ATP assay and NSA data gathered during MSL assembly, test and launch operations (ATLO), it was presumed that the use of the ATP assay could potentially be expanded to support planetary protection bioburden requirements verification on future missions. The objective of the study presented here was to mine and analyze the NSA and ATP data collected during the MSL planetary protection campaign to enable further consideration of the suitability of the ATP-based assay to measure the biological cleanliness of spacecraft surfaces. Furthermore, from a planetary protection perspective, this MSL-based study independently analyzed the data to determine whether or not a “clean” NSA spore count estimation consistently yielded an equivalent “clean” ATP result.

## Materials and methods

### Sample collection

Spacecraft surfaces were sampled during the MSL ATLO mission phase, at the Jet Propulsion Laboratory’s (JPL) Spacecraft Assembly Facility in Pasadena, CA and the Payload Hazardous Servicing Facility at Kennedy Space Center (KSC) in Cape Canaveral, FL. Samples for both spore and ATP assays were collected prior to critical spacecraft assembly events (e.g., environmental thermal vacuum, acoustic, and vibrational testing; transportation of the spacecraft from the primary assembly clean room; hardware rework; hardware last access; and NASA Planetary Protection Officer verification sampling). NSA samples were from small surface areas (25 cm^2^) using cotton swabs (6″ cotton tip applicators, 806-WC, Puritan Medical, Guilford, ME) and from larger surface areas (0.1–1.0 m^2^) using polyester wipes (9″ × 9″ ITW Alpha polyester wipes, Texwipe TX3211, Kernersville, NC), per NASA-HBK-6022 (NASA 2010). In contrast, ATP assay samples were collected only from small surface areas (25 cm^2^) using polyester swabs (Alpha Swab with Long Handle, Texwipe TX761, Kernersville, NC), per NASA-HBK-6022 (Kern et al. 2005; Venkateswaran et al. 2003).

### Sample processing for the NSA

Prior to sample collection, all wipes were folded, rolled, and placed into 50 mL Falcon tubes (Cat No. 352070, BD Biosciences, San Jose, CA) where 15 mL of sterile water was added and then autoclaved. Following this, wipes were aseptically removed from tubes with sterile-gloves. Wipes were then unrolled and folded into quarters such that sampling surfaces were approximately 1/8 the total surface area of the wipe. Sample-laden wipes were then placed in sterile 500 mL glass bottles, which were transported to the laboratory. In most instances, the NSA was carried out immediately. However, due to multiple shift operations and changes in the assembly schedule, samples were occasionally refrigerated at 4 °C for no more than 24 h from the time of collection, before analysis.

After sample collection, 200 mL of sterile rinse solution (85 mg/L potassium dihydrogen phosphate, 200 mg/L Tween 80; pH 7.2) was added to each wipe-containing bottle. When cotton swabs were employed, the pre-sterilized material was removed from the package and pre-moistened with sterile water before the spacecraft surface was sampled. Once a sample was collected, the swab head was excised and placed into 10 mL of sterile water. The sampled wipe or swab was then subjected to vortex mixing at maximum power for 5 s and sonication at 19–27 kHz for 2 min ± 5 s. Samples were then subjected to heat shock at 80 °C ± 2 °C for 15 min.

Suitable aliquots (2 mL each replicates; n = 4) from the heat-shocked and processed samples were aseptically placed in sterile petri dishes to which sterile molten (55 °C) trypticase soy agar (BD Co., Franklin Lakes, NJ) was added. Plates were incubated aerobically at 32 °C in an inverted position, and colony-forming units (CFUs) were enumerated following 24, 48, and 72 h of growth. Appropriate field blanks were performed using sterile wipes or swabs exposed to the clean room environment, in the proximity of the spacecraft, for approximately 1 min. A field blank was collected every 6th wipe or 10th swab. Negative media controls (no sample) and positive media controls (using *Bacillus subtilis* var. niger) were included for each batch of agar.

### Sample processing for the ATP assay

Prior to sample collection, all polyester swabs were pre-sterilized and pre-moistened with 3 mL of sterile molecular-grade water (W4502, Sigma Aldrich, St. Louis, MO) before the spacecraft surface was sampled. Immediately after sample collection, swab heads were cut with pre-sterilized wire cutters and placed into 3 mL of sterile molecular-grade water (Sigma). Swab heads were then immediately taken to the laboratory where the ATP assay was conducted, or were placed in the refrigerator at 4 °C for <2 h. A Check-Light HS Set kit was used, in accordance with manufacturer instructions (Kikkoman Corporation, Noda City, Japan). Sample collection tubes containing the polyester swabs were thoroughly mixed using vortex to release any attached microbial cells. The total ATP content was measured in replicates (a minimum of three per sample) using well-established procedures (Venkateswaran et al. 2003). The ATP assay employed 0.1 mL of sample and 0.1 mL of a detergent solution for cell lysis. The lysis solution provided by the manufacturer contained benzalkonium chloride and a proprietary ATP-releasing agent. The mixture was incubated at room temperature for 1 min, and then 0.1 mL of luciferin-luciferase reagent was added. The sample was then vortexed and bioluminescence measured with a luminometer (Lumitester K-200, Kikkoman Corporation). For each set of assays a standard curve using pure ATP (Sigma, St. Louis, MO) in serial dilutions was carried out to overcome any experimental variances, operator differences, and instrument discrepancies, etc. To ensure the most accurate results, new reagents, disposables, and water were used for each ATP processing event. Negative controls of water were included in all experiments. Field blanks were conducted and processed after every 10 spacecraft samples, where a sterile swab was exposed to the clean room environment but not actively brought in contact with spacecraft surfaces. Samples used for ATP analysis were not subjected to heat shock or sonication as compared to those of the NSA.

### Data mining of MSL NSA and ATP assay results

For MSL’s planetary protection implementation, the 72 h spore counts derived from the NSA were statistically treated to calculate bioburden density (Beaudet 2013). The ATP measurements were treated using an Excel-based macro template that conducts a regression analysis on the standard curve, the average relative luminescence unit (RLU), standard deviation, coefficient of variation. Background noise from the sterile water was subtracted from the average (RLU) before calculating total ATP content, which was carried out by fitting the average RLU per sample from at least three replicates on a standard curve from serial dilutions of an ATP standard (Sigma-Aldrich ATP 0.1 M, 0.5 mL solution) ranging from 1 × 10^−11^ to 1 × 10^−6^ mol. The amount of ATP in units of mmole/sample was then established by multiplying the calculated ATP concentration by the total sample volume. Individual ATP samples were then grouped into broad spacecraft “zones.” Zones were based on the broader spacecraft subsystems representing several larger flight system and launch vehicle components, such as the backshell, descent stage, heatshield, and rover (Benardini et al. 2014). Finally, averages for bioburden densities and ATP mmole/zones were calculated for the specified spacecraft zones.

To compare the cleanliness measured by the NSA (spore burden) and ATP assay (overall microbial burden), only samples collected from adjacent spacecraft surface locations could be used. Unlike the MER-ATP study, aliquots from the same samples were not used to generate ATP assay data from MSL because of MSL’s required standard flight sampling strategy and sample processing. The MSL campaign NSA and ATP datasets were compared side-by-side to correlate the samples using the sampling date and hardware location. The data from the remaining non-adjacent samples were discarded. The data that could be correlated were organized into assays that contributed directly to the MSL bioburden requirement input (deemed accountable bioburden) and assays that were collected from support assays to gauge the spacecraft recontamination potential either from early stage ATLO hardware (i.e. engineering models, fit checks, and/or in a non-flight configuration) or ground support equipment (deemed non-accountable bioburden). Differences in project cleaning practices, microbial reduction, and bioburden requirements made it important to parse and analyze accountable and non-accountable bioburden separately at the highest level of sample binning.

### Data analysis

**Fig. 1:**
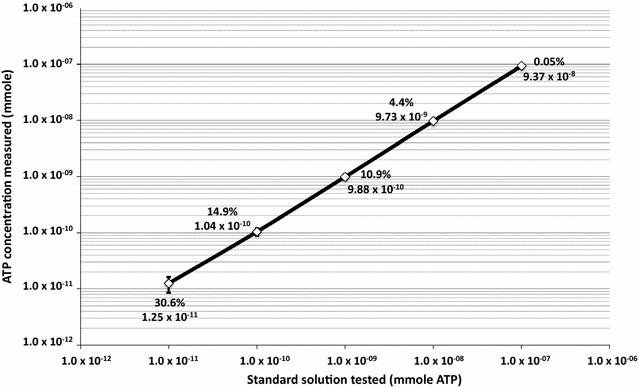
Instrument detection sensitivity of the ATP assay shown as a calibration (or standard) curve. Serially diluted ATP standard solutions were used and 15 measurements in replicates of four for each ATP concentration (n = 60) were carried out. A regression analysis using MS Excel showed r^2^ value as 0.99999. The coefficient of variance is shown in percentage. The ATP derived value (mmole ATP) from the standard curve based on RLU for each serially diluted ATP standard solutions are depicted. The ATP chemicals purchased from Sigma was serially diluted and frozen at −80 °C in multiple replicates. Whenever ATP assay was carried out one tube each of the dynamic range (1 × 10^−11^ to 1 × 10^−6^ mol) of detection limit of the instrument was thawed and used to generate calibration (standard) curve. For each samples of opportunity, fresh set of ATP standard solutions were used and the previously thawed solutions were discarded

A computational analysis was conducted for the data obtained from 320 NSA and 297 ATP samples collected from adjacent locations on MSL hardware. The relevant samples from each dataset were further organized by sampling date and sampling locations, then grouped accordingly into spacecraft “zones”. Notably, multiple combinations of NSA wipes and swabs were used to represent ATP samples, thus the absence of a 1:1 comparison. The analysis directly correlated the bioburden densities and ATP mmole for each spacecraft zone average bioburden densities and ATP mmole/sample for each specified spacecraft zone. For the purposes of this study, a contamination event is defined as ≥1 spore observed during the analysis of a swab or wipe. Similarly, based on the MER-ATP study, as well as the calibration curve generated during this study, a threshold level based on ATP content was suggested as “contamination event.” When analyzing the MSL data, any sample that was <50 RLU (<1.16 × 10^−11^ ATP mmole/sample) was considered as “clean.” Furthermore, as shown in Fig. 1, samples that exhibited <50 RLU were highly variable (>15% coefficient of variance) and not statistically reliable.

## Results

Of the 4853 NSA samples and 606 ATP samples collected during the MSL ATLO campaign and analyzed here, only 320 NSA samples and 297 ATP samples were collected from adjacent locations. The other samples had to be excluded from further consideration for this analysis, since they could not be directly compared. Samples collected were from a variety of surfaces to accurately capture the cleanliness of spacecraft subsystems, or a given component. Sample characteristics are provided in Table 1. Forty-eight samples deemed “non-accountable” were collected from the descent stage (early MSL ATLO phase); the rover interior filters [inside chambers that communicate with the external environment only through high efficiency, particulate air (HEPA, removes 99.97% of all particles >0.3 Âµm)]; and the launch vehicle isolation diaphragm (support hardware that maintains adequate hardware spore cleanliness levels greater than flight hardware requirements). These non-accountable samples were collected and processed for reasons related to monitoring cleanliness, but were not used in calculating MSL’s overall bioburden density. The characteristics of actual flight hardware samples, 272 in total, whose bioburden densities were accounted, are also provided in Table 1.

**Table 1:** Spacecraft surface characteristics sampled during this study

Spacecraft surface	Sample location	Final accountable sample	Zone name	Date	Spacecraft surface sampled	Number of samples tested for NSA	Number of samples tested for ATP
*Flight samples*							
Backshell and balance mass device	KSC	Y	Backshell	6/6/11	15	10	10
Spacecraft stack	KSC	Y	Backshell	9/20/11	38	4	9
Spacecraft stack prior to encapsulation	KSC	Y	Backshell	10/8/11	16	6	2
Descent pre-closeout	KSC	Y	Descent stage	8/12/11	37	94	32
Spacecraft stack	KSC	Y	Descent stage	9/16/11	72	41	62
Descent stage electronics	JPL	Y	Descent Stage	4/5/11	7	6	6
Spacecraft stack prior to encapsulation	KSC	Y	Heatshield	10/8/11	21	6	21
Rover pre-closeout	KSC	Y	Rover	8/10/11	53	31	34
Spacecraft stack	KSC	Y	Rover	9/20/11	70	54	55
Spacecraft stack prior to encapsulation	KSC	Y	Rover	10/8/11	23	20	18
*Non-flight samples*							
Descent stage	JPL	N	Descent stage	3/7/08	20	20	20
Rover interior	JPL	N	Rover	5/20/08	18	18	18
Launch vehicle isolation diaphragm	KSC	N	Non-flight	10/27/11	10	10	10
Total number of samples analyzed for accountable flight assays					400	320	297

### Spore burden of MSL surfaces

Among 272 accountable MSL spacecraft surface samples subjected to the estimation of spore burden using NSA, 22 samples (~8% of the 272 MSL surface samples) showed the presence of at least one spore. When all 272 samples were grouped into zones, only ~3% of the descent stage (five of 141 samples) and rover (three of 105 samples) surface samples were shown to be dirty (≥1 spore) compared to 40% of the backshell (eight of 20 samples) and 100% of the heatshield (6 of 6 samples) components (Table 2). When a larger surface area was sampled (1 m^2^) using polyester wipes, spore contamination (one to 26 spores per wipe) was observed in 19 out of 67 wipes (~28%). When NASA-certified cotton swabs were used to sample smaller (25 cm^2^) spacecraft surfaces, only three out of 205 samples showed the presence of spores and none exhibited more than one spore per sample. In contrast to accountable MSL surfaces, non-accountable surfaces yielded 15-fold more spores using swabs and 2.2-fold more spores using wipes. In summary, the NSA side-by-side samples utilized for this analysis represented a 47.77 m^2^ spacecraft surface area and exhibited 439 spores, equivalent to 9.2 spores per m^2^. This spore burden value is less than the allowable 300 spores per m^2^ limit (NASA 2011) and less than the observed average MSL bioburden density of 22 spores per m^2^ (Benardini et al. 2014).

**Table 2:** Spacecraft zone-wise comparison about the different bioburden assays

Zone Name	ATP assay			NASA standard spore assay			
	# of samples tested using swabs	Percent clean samples (<2.33 × 10^−11^ mmol ATP)	Percent dirty samples (≥2.33 × 10^−11^ mmol ATP)	# of samples tested using:		Percent clean samples (<1 spore)	Percent dirty samples (≥1 spore)
				Swabs	Wipes		
Backshell	21 (4)^a^	81.0	19.0	9 (1)	11 (7)	60.0	40.0
Descent stage	100 (9)	91.0	9.0	109 (1)	32 (4)	96.5	3.5
Heatshield	21 (7)	66.7	33.3	–	6 (6)	–	100.0
Rover	107 (15)	86.0	14.0	87 (1)	18 (2)	978.1	2.8

^a^Numeral in parentheses denotes # of samples exhibiting more than the threshold level of cleanliness

### ATP assay-based cleanliness of MSL surfaces

In total, 249 of the accountable MSL spacecraft surface samples that were measured for ATP content were used for the comparative analysis reported here. Of the 249 samples, 35 (~15% of the MSL surface samples) showed >2.3 × 10^−11^ mmol ATP per 25 cm^2^ surface area. Similar to the NSA results, when all the 249 samples were grouped in zones, the descent stage (9% dirty) and rover (14% dirty) surface samples were determined to be cleaner than backshell (19% dirty) and heatshield (~33% dirty) surface samples (Table 2).

The luminometer instrument used to measure ATP reproducibly detected ≥50 RLU (equivalent to ≥1.6 × 10^−11^ ATP mmoles per sample). The coefficient of variance was <15% for those samples that exhibited a >50 RLU reading. The calibration curve of the instrument was linear (r^2^ = 0.999) when a known amount of ATP, ranging from 1.0 × 10^−11^ to 1.0 × 10^−7^ mmol (Fig. 1), was used. The dynamic range of the instrument was 50 RLU to 500,000 RLU.

**Fig. 2:**
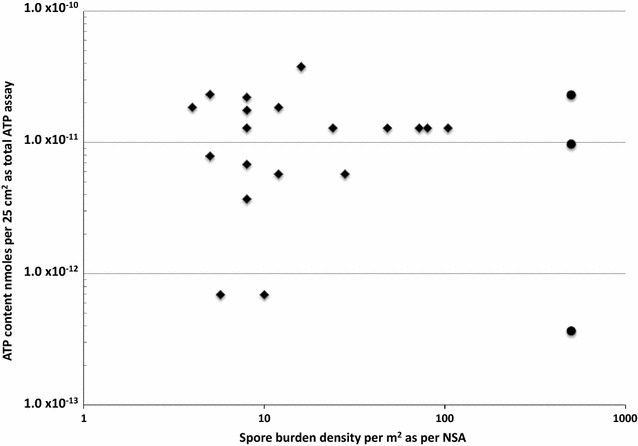
Comparison of NSA and ATP assay in measuring cleanliness of MSL spacecraft accountable-surface samples for 22 out of 272 samples. Swab samples for NSA are represented by circle and wipes are shown in diamonds. Total number of spacecraft flight samples tested by NSA were 272, of which 67 were wipes and 205 were swabs. In total only 22 of the NSA samples exhibited ≥1 spore; the 250 samples that exhibited no spores are not shown. Spore counts (CFU) and ATP values (RLU) of all surfaces are provided as supplementary file (Additional file 1: Table S1)

The ATP assay could not be performed using aliquots of the same samples collected for the NSA because of stringent MSL planetary protection protocols, as well as mission schedule conflicts. As a result, a direct comparison in the analysis reported here was not possible. However, samples from numerous side-by-side samples (~250 side-by-side samples) were employed for the cultivation of spores and measurement of ATP content. Thus, it was still possible to perform a valid comparison using samples from adjacent surfaces. With this assumption, spore bioburden density was plotted against ATP content (Fig. 2). Since majority of the samples (>85%) had no spore counts only 22 samples that showed presence of at least 1 or more spores were depicted in Fig. 2. Among these 22 samples only 4 samples possessed ≥1 spore and were recommended for further cleaning (>2.3 × 10^−11^ ATP mmoles).

## Discussion

The focus of ATP sampling for MSL was to monitor the microbial cleanliness of spacecraft surfaces that underwent microbial reduction and were assembled in controlled, HEPA-filtered clean rooms. In general, spacecraft components were subjected to one or more of the following microbial reduction processes or practices: precision cleaning consisting of an alkaline detergent and mechanical scrubbing followed by sonication in a series of solvents (JPL 1990), isopropyl alcohol wiping (Benardini et al. 2014), hardware covering with appropriate draping to prevent recontamination, or dry heat microbial reduction (Benardini et al. 2014). The HEPA-filtered air and any accumulated particulates on the spacecraft, ground support equipment, and floor of the humidity-controlled, desiccated clean room surfaces provide low quantities of nutrients to inhabiting microbes (Venkateswaran et al. 2001). Particulate materials from spacecraft surfaces within HEPA-filtered environments showed a low incidence of spores and cultivable microbial populations (Ghosh et al. 2010; La Duc et al. 2004, 2007). Upon examination using state-of-the art molecular microbial approaches an equal representation of both Gram-negative and Gram-positive microbial populations were revealed, most of them were viable but yet to be cultivated (La Duc et al. 2003, 2009; Vaishampayan et al. 2010; Venkateswaran et al. 2001, 2012). In total, given the low cultivable spore counts in conjunction with the extremely low ATP levels the MSL ATLO campaign results supports these earlier findings illustrating the cleanliness of spacecraft surfaces.

When microbial reduction processes and practices were stringently performed, and subsystems were assembled in a nutrient-deprived class 100 K or better clean room, only 15% of the 249 samples analyzed for this study exceeded the 100 RLU (2.33 × 10^−11^ ATP mmoles per 25 cm^2^) threshold cleanliness limit used by MSL. This threshold level was consistent with the previous MER-ATP study where surfaces with <2.57 × 10^−11^ ATP mmoles per 25 cm^2^ were only recommended for optional cleaning, whereas surfaces surpassing >3.51 × 10^−11^ ATP mmoles per 25 cm^2^ were required to be cleaned again. (Kern et al. 2005).

This postlaunch analysis of MSL ATP data also supported MER-ATP conclusions, where it was noted that ATP was a useful biomarker for measuring spacecraft surface cleanliness (Kern et al. 2005). The analysis of MSL data expands the ATP knowledgebase in that it demonstrates the application of ATP threshold established from the MER-ATP study when applied to a flight project, i.e., not in a controlled experimental setting. When applying binary threshold levels for acceptable cleanliness, as in the MSL case, a clean ATP assay (<2.33 × 10^−11^ ATP mmoles per 25 cm^2^) result also yielded a clean NSA (0 spore) result, demonstrating the same observable planetary protection outcome with high confidence (~97%, 242 out of 250 samples; Fig. 2). Likewise, when spacecraft surface samples did not exceed the binary ATP threshold, they correlated with the clean NSA results and within acceptable MSL flight processing limits. During this analysis, ~98.5% of samples (268 out of 272 samples from accountable surface areas) were shown to be clean by both the NSA and ATP assay. Therefore, for future missions seeking a binary cleaning threshold, the threshold value used by MSL during ATLO (<2.33 × 10^−11^ ATP mmoles per 25 cm^2^) could be utilized to assess the cleanliness of hardware during the final stages of spacecraft assembly. These data also strongly support the notion that the ATP benchmark demonstrates the same planetary protection “clean” outcome as NSA swabs on flight hardware that have undergone multiple microbial reduction practices and processes (heat microbial reduction, alcohol cleaning, etc.).

The distinction between the examination of microbial burden on accountable and non-accountable surfaces proved to be of great importance in the data analysis. Unlike accountable spacecraft surfaces that have undergone microbial reduction, it was not possible to consistently evaluate non-accountable surfaces, which were not cleaned or maintained in the same way as flight hardware, using the threshold ATP cleanliness limit as an alternative to the spore assay. In many samples from non-accountable surfaces, notably before the final stage of spacecraft assembly, ATP levels were relatively high (30% of the samples). This is probably attributable to the higher bioburden levels allowed for the launch vehicle (500–1000 spores m^2^) (Benardini et al. 2014), the presence of non-spore-forming cells (Venkateswaran et al. 2003), or lysed but dead cells (Ghosh et al. 2010; La Duc et al. 2007) that might not have been adequately removed.

In summary, the ATP assay allowed MSL project personnel to make informed decisions regarding spacecraft surface cleanliness within minutes versus three days using the NSA, which was of significant benefit to the schedule. When necessary, based on the measured ATP levels, additional cleaning could be done almost immediately (~1 h from sampling) as hardware was assembled, with resampling being conducted either later on the same day or the following day. The implementation of the ATP assay provided a means for the project to rapidly assess spacecraft cleanliness and allowed the project to decrease the risk of potential disassembly, recleaning, and reassembly of the hardware in the event of a NSA dirty sample. The application of the ATP assay on MSL demonstrates the value of alternative microbial methods for process/system control, optimization, and routine monitoring of the general microbial quality of spacecraft subsystems during assembly process.

This analysis of MSL ATLO data was conducted independently of the MSL planetary protection campaign and the required spore burden verification assessments for the MSL mission. However, it did identify certain circumstances and conditions in which there was excellent alignment of the cleanliness information from the ATP assay and the NSA in assessing surface cleanliness of hardware being prepared for flight.

## Additional file

**Additional file 1: Table S1.** Detailed biological contamination range with respect to ATP molecule and spore counts as measured by NASA Standard Spore Assay.
